# Clinical trial evidence on emerging pharmacological therapies for hypoactive sexual desire disorder in women: a systematic review and analysis of completed studies registered on ClinicalTrials.gov

**DOI:** 10.3389/fmed.2026.1789809

**Published:** 2026-05-22

**Authors:** Ahmed M. Ashour

**Affiliations:** Department of Pharmacology and Toxicology, College of Pharmacy, Umm Al-Qura University, Makkah, Saudi Arabia

**Keywords:** ClinicalTrials.gov, female sexual health, hypoactive sexual desire disorder, pharmacological therapy, sexual desire, women’s health

## Abstract

**Background:**

Hypoactive Sexual Desire Disorder (HSDD) is a prevalent and distressing condition affecting adult women and is associated with significant impairments in quality of life and interpersonal relationships. Despite increasing recognition of female sexual health as a clinical priority, pharmacological treatment options for HSDD remain limited, and evidence from individual clinical trials remains heterogeneous. Therefore, a systematic evaluation of registered clinical trials is essential to better understand therapeutic development, efficacy endpoints, and safety profiles of emerging treatments.

**Methods:**

This systematic review was conducted in accordance with PRISMA 2020 guidelines using a structured search of ClinicalTrials.gov. Completed interventional clinical trials evaluating pharmacological therapies for HSDD in adult women were identified using predefined eligibility criteria. Data were extracted on study design, participant characteristics, investigational agents, efficacy outcomes related to sexual desire and distress, and reported safety outcomes. Findings were synthesized descriptively without pooled quantitative analysis.

**Results:**

A total of nine completed pharmacological clinical trials met the inclusion criteria. Most were Phase II and Phase III randomized, double-blind, placebo-controlled studies, primarily enrolling premenopausal women with acquired, generalized HSDD. Investigational therapies predominantly targeted central nervous system pathways, with flibanserin and bremelanotide representing the most extensively studied agents. Efficacy outcomes commonly included validated patient-reported measures such as the Female Sexual Function Index desire domain and the Female Sexual Distress Scale; however, endpoint designation and reporting completeness varied across trials. Safety data were inconsistently reported, with adverse events reflecting the pharmacological mechanisms of the agents studied.

**Conclusion:**

The findings highlight both progress and persistent gaps in the pharmacological treatment landscape for HSDD. Variability in trial design, outcome measures, and reporting practices limits cross-trial comparison and clinical interpretation. Further research with standardized methodologies and comprehensive reporting is needed to strengthen the evidence base.

## Introduction

Hypoactive Sexual Desire Disorder (HSDD) is a prevalent yet underrecognized condition affecting adult women. It is primarily characterized by a persistent or recurrent deficiency or absence of sexual desire accompanied by marked personal distress or interpersonal difficulty (1–3). Classified under Female Sexual Interest/Arousal Disorder (FSIAD) in the Diagnostic and Statistical Manual of Mental Disorders, Fifth Edition (DSM-5), HSDD represents a complex biopsychosocial condition influenced by neurobiological, hormonal, psychological, and sociocultural factors (4, 5). Despite its substantial impact on quality of life, emotional well-being, and intimate relationships, female sexual desire disorders have historically received limited clinical and research attention compared with male sexual dysfunction (6, 7). An important observation from the current literature is that despite its clinical relevance, the evidence base for HSDD remains fragmented, with limited synthesis of how clinical trials are designed, conducted, and reported across this therapeutic area.

Epidemiological studies suggest that reduced sexual desire is one of the most commonly reported sexual concerns among women, with prevalence estimates ranging from 22.7% to over 72.2% in premenopausal women and in the range of 37.3 to 78.2% in perimenopausal women (8). Importantly, not all women with low sexual desire meet the criteria for HSDD, notably as distress is a defining component of the diagnosis (9). This distinction highlights the need for rigorously designed clinical trials that not only assess changes in sexual desire but also evaluate associated distress and functional outcomes. However, variability in diagnostic definitions, outcome measures, and study designs have complicated the interpretation and comparison of findings across trials. Viewed in context, this heterogeneity strongly indicates a need for structured evaluations that move beyond individual trial findings and instead examine broader patterns in study design, endpoint selection, and reporting practices.

Pharmacological treatment options for HSDD remain limited (2, 10, 11). Currently, approved therapies target central nervous system pathways implicated in sexual motivation and reward, rather than peripheral genital arousal mechanisms (11). Agents, particularly flibanserin and bremelanotide, represent significant advances, yet their modest efficacy, safety concerns, and restrictive prescribing requirements have fueled ongoing research into novel pharmacological approaches (12–14). As a result, multiple investigational therapies targeting serotonergic, dopaminergic, melanocortin, and hormonal pathways have been evaluated in clinical trials over the past decade (13). Notably, it is vital to understand the scope, design, and outcomes of these trials for the purpose of contextualizing current treatment options and guiding future drug development. Importantly, registry-based analyses provide access to both published and unpublished studies, thereby reducing the risk of publication bias and enabling a more comprehensive assessment of the clinical research landscape. To date and to the best of our knowledge upon preliminary review of literature, no study has systematically examined registered clinical trials for HSDD with a focus on trial characteristics, outcome measures, and safety reporting patterns.

ClinicalTrials.gov serves as the largest publicly accessible registry of clinical studies worldwide and plays a critical role in promoting transparency, accountability, and reproducibility in clinical research. Since the implementation of trial registration requirements, the database has become an invaluable resource for examining research trends, methodological rigor, and reporting practices across therapeutic areas. Closer in connection to the current study, for conditions such as HSDD that have not seen much published literature, registry-based analyses serve to provide a more comprehensive view of the clinical trial outlook including completed studies that may not have resulted in peer-reviewed publications. Despite the growing number of registered trials evaluating pharmacological interventions for HSDD, there remains a lack of consolidated analyses examining how these studies are designed, which efficacy and safety endpoints are prioritized, and what patterns emerge across investigational agents (15, 16). Existing reviews, often, focus on individual drugs or summarize published outcomes without systematically assessing trial characteristics or registry-reported data. Accordingly, this critical gap limits the ability of clinicians, researchers, and policymakers to critically appraise the robustness of the evidence base and to identify methodological shortcomings that may hinder translation into clinical practice.

Moreover, heterogeneity in outcome measures presents a significant challenge in HSDD research. In this context, a review of existing literature shows that trials commonly employ validated instruments such as the Female Sexual Function Index (FSFI) desire domain and the Female Sexual Distress Scale (FSDS) (17–19), yet differences in endpoint selection, timing of assessments, and definitions of clinically meaningful change complicate cross-trial comparisons. Safety reporting also varies, particularly for centrally acting agents associated with neurological, cardiovascular, or gastrointestinal adverse effects (20). Following these observations, it is notable that a systematic examination of how efficacy and safety outcomes are captured and reported in registered trials is of essence and warranted. In addition, improving therapeutic development for HSDD, from a multidisciplinary perspective, calls not only for the identifying of efficacious agents but also addressing broader issues related to trial inclusivity, participant characteristics, and real-world applicability. Factors such as menopausal status, comorbid conditions, concomitant medication use, and psychosocial context may influence treatment response in HSDD cases yet are inconsistently reported across studies (21, 22). Evaluating the extent to which clinical trials account for these variables, in our view, is important as it will help inform more patient-centered and clinically relevant research designs. Against this backdrop, in the present study, our focal aim was to analyze completed interventional clinical trials registered on ClinicalTrials.gov that evaluated pharmacological therapies for HSDD in adult women. Our analysis focused on study design, participant characteristics, efficacy endpoints related to sexual desire and distress, and reported safety outcomes, with the objective of identifying patterns, methodological strengths, and gaps in the current evidence base. Rather than assessing pooled efficacy, our focus was primarily on the identification of patterns, strengths, limitations, and gaps in the existing evidence base.

## Methodology

### Study design

In order to attain the primary objective, the current study employed a descriptive analytical design based on the systematic extraction and synthesis of data from completed interventional clinical trials registered on ClinicalTrials.gov. In the data analysis, we focused on pharmacological therapies evaluated for the treatment of HSDD in adult women. No individual patient-level data were accessed, and all information was derived from publicly available trial registry records to ensure adherence to ethical essentials in research.

### Reporting guideline

This systematic review was conducted and reported in accordance with the Preferred Reporting Items for Systematic Reviews and Meta-Analyses (PRISMA) 2020 statement.

### Data source and search strategy

Clinical trial data were obtained exclusively from ClinicalTrials.gov through a structured search which we conducted to identify relevant trials for inclusion. The researchers used the following criteria to identify and analyze data from qualifying clinical trial studies. The first item was condition or disease terms where we used *Hypoactive Sexual Desire Disorder*, *Female Sexual Interest/Arousal Disorder* as the keywords. Next, we focused the search in terms of type and included *Interventional studies* only. The third item in the search criteria was recruitment status where we included only completed trials. We also had female gender as another criteria item, and in terms of age our search focused on adults, particular those of at least 18 years. Further, we limited to studies evaluating pharmacological interventions, but did not have restrictions on geographic location or year of trial registration. The final search was conducted on December 27, 2025.

### Systematic review protocol and registration

A formal review protocol was not registered in a publicly accessible database such as PROSPERO. However, the methodology for this systematic review, including the eligibility criteria, search strategy, and data extraction approach, was predefined prior to data collection and consistently applied throughout the review process.

### Eligibility criteria

#### Inclusion criteria

Trials were eligible for inclusion if they were interventional clinical trials focused solely on evaluating pharmacological therapies intended to treat HSDD or closely related diagnostic entities in adult women, enrollment of female participants aged 18 years or older and completed study status at the time of data extraction. Further, we included trials that had availability of outcome measures that were related to sexual desire and or sexual distress, and were either Phase II, III, or IV trials.

#### Research question and PICOS framework

This systematic review aimed to address the following research question: *What are the characteristics, efficacy outcomes, and safety profiles of pharmacological therapies evaluated in completed interventional clinical trials for hypoactive sexual desire disorder (HSDD) in adult women?*

The research question was structured using the PICOS framework as follows: Population (P): adult women aged 18 years or older diagnosed with HSDD or closely related diagnostic classifications; Intervention (I): pharmacological therapies intended for the treatment of HSDD; Comparator (C): placebo or active comparator, where applicable; Outcomes (O): efficacy outcomes related to sexual desire and associated distress (e.g., Female Sexual Function Index desire domain and Female Sexual Distress Scale or related instruments), as well as safety and tolerability outcomes; and Study design (S): completed interventional clinical trials (Phase II–IV) registered on ClinicalTrials.gov.

#### Exclusion criteria

Trials were excluded if they were observational, registry-only, or non-interventional in design, evaluated non-pharmacological interventions exclusively or those which included male participants only or did not report female-specific outcomes. Also, studies that focused on sexual dysfunction secondary to other primary medical conditions without specific assessment of HSDD or those that lacked sufficient methodological or outcome information in the registry record were excluded.

### Study selection process

**Figure 1:**
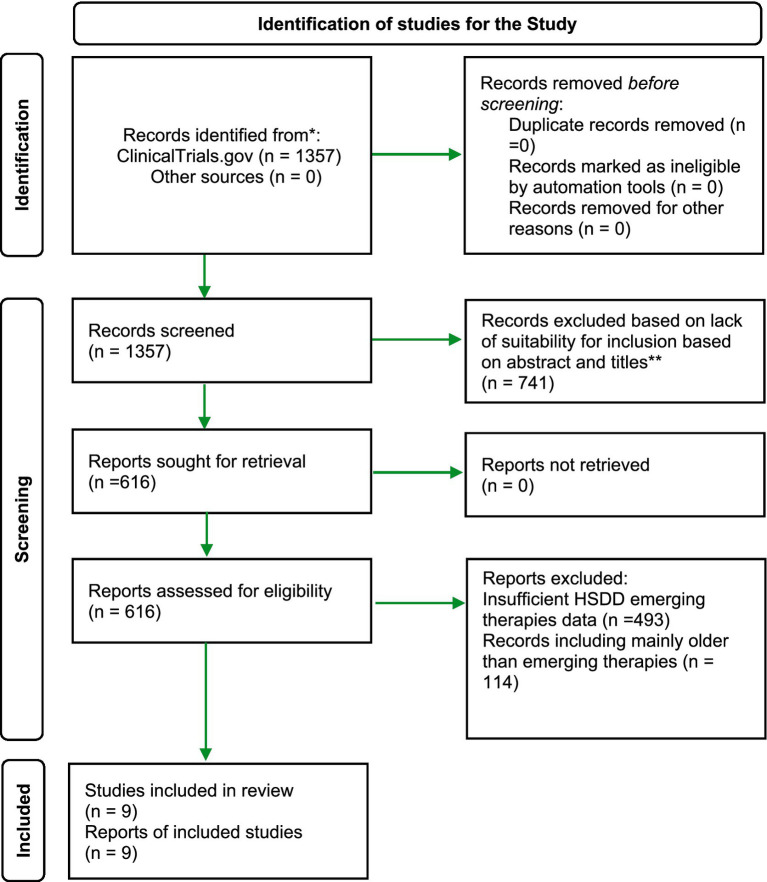
PRISMA flow chart for trials selection from Clinicaltrials.gov (search conducted on December 27, 2025).

All records retrieved from the initial search were screened for eligibility based on trial titles and registry descriptions. Full registry entries of potentially eligible trials were then reviewed to confirm inclusion criteria. Reasons for exclusion were documented at each stage of screening. The study identification and selection process are summarized in Figure 1 flow diagram illustrating the number of records identified, screened, excluded, and included in the final analysis.

### Data extraction

Data were independently extracted from each eligible trial registry entry using a standardized data collection framework and the variables we extracted included trial characteristics, particularly the clinical trial phase, study design identifying whether it was a randomization, blinding, or comparator type, sample size and enrollment status, study duration and follow-up period, and geographic region(s) of recruitment. We also extracted data relating to participant characteristics, the core items here being age, menopausal status, diagnostic criteria used for HSDD, and baseline sexual function or distress scores. On interventions, we extracted data relating to investigational pharmacological agent, dosage and route of administration, and comparator (placebo or active control).

Other two important areas we collected data were efficacy and safety outcomes. On efficacy, we extracted data on primary and secondary efficacy endpoints, sexual desire measures, particularly on Female Sexual Function Index desire domain, sexual distress measures, and timing of outcome assessments. Regarding safety outcomes, we collected data on reported adverse events, serious adverse events, treatment discontinuations due to adverse events, as well as safety monitoring parameters in trials where these were reported.

### Data synthesis and analysis

Data were synthesized using descriptive analytical methods. Trial characteristics, outcome measures, and safety data were summarized using frequencies, proportions, and narrative descriptions. Given the heterogeneity of study designs, interventions, and outcome measures, no quantitative meta-analysis was conducted. Comparative patterns across pharmacological classes and trial phases were examined qualitatively to identify trends in efficacy reporting, safety profiles, and methodological approaches. Where available, consistency or variability in outcome definitions and assessment tools was noted.

### Risk of bias assessment

A formal risk of bias assessment using tools such as the Cochrane Risk of Bias instrument was not undertaken. This decision was based on the registry-based nature of the review, as the analysis relied exclusively on information available in ClinicalTrials.gov records. Many registry entries lacked sufficient methodological detail and complete outcome reporting required for reliable domain-level risk of bias evaluation. Consequently, risk of bias was considered narratively by examining key trial characteristics, including study design, randomization, blinding, comparator type, and completeness of reported results.

### Ethical considerations

This study analyzed data from publicly available clinical trial registry records and did not involve access to individual-level patient data. As such, institutional review board approval and informed consent were not required.

## Results

### Study selection

The initial search of ClinicalTrials.gov identified a total of 1,357 records related to HSDD. After screening titles and registry descriptions, 741 trials were excluded due to non-interventional design, non-pharmacological interventions, and for ineligible populations. Full registry entries of 616 trials were reviewed in detail, resulting in 9 completed interventional studies meeting the inclusion criteria. The PRISMA chart summarizing the study identification and selection process is presented in Figure 1.

### Characteristics of included studies

The included trials consisted primarily of Phase II and Phase III studies, with one Phase IV study focusing on mechanistic or post-approval evaluation. Most trials employed a randomized, double-blind, placebo-controlled design, consistent with regulatory standards for efficacy assessment in sexual medicine. One trial represented an open-label safety extension assessing longer-term tolerability. In terms of sample sizes, the included trials varied substantially as these ranged from early-phase dose-finding studies enrolling fewer than 400 participants to large multicenter Phase III trials enrolling over 1,000 participants. Also, study durations were most commonly 12 to 24 weeks with one long-term safety extension lasting 52 weeks. Longer-term efficacy follow-up beyond 6 months was infrequently reported (see Table 1).

**Table 1:** Summary of data from ClinicalTrials.gov (search conducted on December 27, 2025).

Trial identifier (NCT)	Study phase	Study design	Sample size (*n*)	Population characteristics	Investigational agent	Comparator	Primary efficacy outcomes	Study duration
NCT00491829	Phase III	Randomized, double-blind, placebo-controlled	945	Premenopausal women with acquired, generalized HSDD	Flibanserin (50 mg & 100 mg)	Placebo	FSFI-desire score; safety and tolerability	24 weeks
NCT00572029	Phase III	Randomized, double-blind, placebo-controlled	880	Premenopausal women with HSDD	Flibanserin (100 mg)	Placebo	FSFI-desire domain; sexual distress	24 weeks
NCT00654009	Phase III	Randomized, double-blind, placebo-controlled	1,087	Premenopausal women with HSDD	Flibanserin (100 mg)	Placebo	FSFI-desire score; FSDS	24 weeks
NCT00996372	Phase III	Randomized, double-blind, placebo-controlled	Not reported in registry (NR)	Postmenopausal women with HSDD	Flibanserin	Placebo	FSFI-desire score; distress measures	24 weeks
NCT00441558	Phase III	Open-label safety extension	1,723	Women completing prior flibanserin trials	Flibanserin	None	Long-term safety; adverse events	52 weeks
NCT02338960	Phase III	Randomized, double-blind, placebo-controlled	613	Premenopausal women with HSDD	Bremelanotide (PT-141)	Placebo	FSFI-desire; FSDS-DAO Item 13	24 weeks
NCT02333071	Phase III	Randomized, double-blind, placebo-controlled	628	Premenopausal women with HSDD	Bremelanotide (PT-141)	Placebo	FSFI-desire; FSDS-DAO Item 13	24 weeks
NCT01735539	Phase II	Randomized, double-blind, placebo-controlled	327	Premenopausal women with HSDD	Bremelanotide (dose-finding)	Placebo	Change in sexual desire score	12 weeks
NCT04179734	Phase IV	Randomized, mechanistic study	Not reported in registry (NR)	Adult women with HSDD	Bremelanotide (MC4R agonist)	Placebo	Neuroimaging and physiological endpoints	Variable

FSFI, Female Sexual Function Index; FSDS, Female Sexual Distress Scale; FSDS-DAO, Female Sexual Distress Scale–Desire/Arousal/Orgasm; NR, not reported in registry.

### Participant characteristics

All included trials enrolled adult women aged 18 years or older with a diagnosis of HSDD or a closely related classification. The majority of efficacy trials focused on premenopausal women with acquired, generalized HSDD, while a smaller number included postmenopausal women or mixed populations. Where reported, mean participant age typically ranged from the early 30s to mid-50s. Diagnostic criteria varied across trials. Some studies referenced DSM-based definitions, while others relied on investigator-defined criteria supplemented by validated screening instruments. Baseline measures of sexual desire and distress were commonly recorded, although the level of detail available in registry entries varied.

### Investigational pharmacological therapies

The pharmacological agents evaluated across the included trials were predominantly centrally acting therapies targeting neurobiological pathways involved in sexual motivation and reward. Two principal development programs were identified, these being flibanserin, primarily evaluated as a daily oral therapy, and bremelanotide (PT-141) which was investigated as an on-demand subcutaneous agent acting via melanocortin receptor pathways. Comparators were almost exclusively placebo controls, particularly in Phase II and III trials, and active comparators were not commonly employed, reflecting the limited availability of established pharmacological treatments for HSDD during the trial periods.

### Risk of bias considerations

Overall, most included studies were randomized, double-blind, placebo-controlled Phase II and Phase III studies, suggesting a generally low risk of performance and detection bias. However, limitations related to incomplete reporting within ClinicalTrials.gov records were identified. Several completed trials did not provide detailed numerical outcome data or comprehensive adverse event reporting, which may introduce reporting bias and limit interpretability (see Table 2).

**Table 2:** Risk of bias summary of included trials.

Trial ID	Randomized	Blinded	Comparator	Results reported	Key concern	Overall risk
NCT00491829	Yes	Yes	Placebo	Partial	Incomplete numerical outcomes	Moderate
NCT00572029	Yes	Yes	Placebo	Partial	Limited detail in outcomes	Moderate
NCT00654009	Yes	Yes	Placebo	Partial	Incomplete efficacy reporting	Moderate
NCT00996372	Yes	Yes	Placebo	Limited	Missing sample size and details	High
NCT00441558	No (Open-label)	No	None	Partial	No blinding, safety-only design	High
NCT02338960	Yes	Yes	Placebo	Partial	Limited adverse event detail	Moderate
NCT02333071	Yes	Yes	Placebo	Partial	Incomplete reporting of outcomes	Moderate
NCT01735539	Yes	Yes	Placebo	Partial	Dose-finding variability	Moderate
NCT04179734	Yes	Unclear	Placebo	Limited	Mechanistic study, incomplete data	High

### Efficacy outcomes

Efficacy assessment across trials focused on sexual desire and associated distress, though endpoint selection and hierarchy varied. The most frequently reported outcome measures included the Female Sexual Function Index (FSFI) desire domain and the Female Sexual Distress Scale (FSDS) or related derivatives, such as the FSDS-DAO. Trials differed in whether these measures were designated as primary or secondary endpoints and in the timing of outcome assessments. Most studies conducted repeated assessments at baseline and at multiple follow-up visits, commonly at 4, 8, and 12 weeks. Registry entries typically indicated whether primary endpoints were met; however, detailed numerical efficacy data were inconsistently reported. Overall, registry-reported outcomes suggested heterogeneous efficacy across investigational agents, with some trials demonstrating improvements in sexual desire and reductions in distress relative to placebo, while others reported more modest or variable effects. A structured summary of efficacy outcomes and reported safety data across trials is presented in Table 3.

**Table 3:** Summary of efficacy and safety outcomes.

Trial ID	Drug	Primary outcome	Endpoint met	Effect direction	Common adverse events	Serious adverse events	Notes
NCT00491829	Flibanserin	FSFI desire score	Yes	↑ Improvement	Dizziness, nausea	Rare	Standard Phase III trial
NCT00572029	Flibanserin	FSFI desire + distress	Yes	↑ Improvement	CNS-related symptoms	Rare	Consistent with other trials
NCT00654009	Flibanserin	FSFI + FSDS	Yes	↑ Improvement	Neurological, GI symptoms	Rare	Typical efficacy pattern
NCT00996372	Flibanserin	FSFI + distress	Not reported clearly	Unclear	Not fully reported	Not reported	Postmenopausal population
NCT00441558	Flibanserin	Long-term safety	Not applicable	Not applicable	Similar to prior trials	Rare	Open-label extension
NCT02338960	Bremelanotide	FSFI + FSDS-DAO	Yes	↑ Improvement	Nausea, flushing	Rare	On-demand therapy
NCT02333071	Bremelanotide	FSFI + FSDS-DAO	Yes	↑ Improvement	Nausea, headache	Rare	Similar to companion trial
NCT01735539	Bremelanotide	Sexual desire score	Yes	↑ Improvement	Mild to moderate AEs	Rare	Dose-finding study
NCT04179734	Bremelanotide	Mechanistic endpoints	Not applicable	Not applicable	Not reported	Not reported	Imaging/physiological study

Due to incomplete reporting in ClinicalTrials.gov entries, efficacy and safety outcomes are summarized descriptively, and “not reported” indicates absence of detailed data in the registry.

### Safety and tolerability

Safety outcomes were reported for the majority of included trials, though the depth and consistency of reporting varied. Common adverse events reflected the central nervous system mechanisms of the investigational agents and included neurological, gastrointestinal, and cardiovascular-related symptoms. Serious adverse events were uncommon, and treatment discontinuation due to adverse events occurred in a minority of participants. Longer-term safety data were primarily derived from an open-label extension study, which focused on tolerability and adverse event monitoring over a 52-week period. Standardized reporting of adverse event frequency and severity was not uniformly available across registry entries, limiting direct cross-trial comparison.

### Reporting completeness

Several completed trials lacked fully posted results or provided only high-level outcome summaries within ClinicalTrials.gov. This incomplete reporting constrained comprehensive evaluation of efficacy and safety outcomes and highlights ongoing challenges related to result dissemination and transparency in HSDD pharmacotherapy research.

## Discussion

This registry-based analysis aimed at providing a consolidated overview of completed interventional clinical trials evaluating pharmacological therapies for HSDD in adult women. As a descriptive registry-based analysis, this study does not assess comparative efficacy but rather identifies patterns in trial design, outcome selection, and reporting practices. The findings highlight key trends in trial design, therapeutic focus, outcome selection, and reporting practices within the evolving arena of female sexual health pharmacotherapy (23, 24). The findings emphasize both the progress achieved in developing centrally acting treatments for HSDD as well as the persistent methodological and evidentiary challenges that continue to limit clinical translation.

### Principal findings

The analysis identified nine completed pharmacological trials meeting inclusion criteria, the majority of which were Phase II and Phase III randomized, double-blind, placebo-controlled studies. Two primary therapeutic development programs, flibanserin and bremelanotide, accounted for most trials, which is consistent with current literature and reflects the narrow range of pharmacological approaches that have progressed to late-stage clinical evaluation (25). This concentration may in context, reflect a focus on central nervous system–targeted mechanisms, although it may also indicate limited diversification in late-stage pharmacological development.

Most efficacy trials enrolled premenopausal women with acquired, generalized HSDD, while postmenopausal women were underrepresented and, on another dimension, study durations were typically limited to 12–24 weeks, with only one long-term extension study providing safety data beyond 6 months. These patterns indicate that current evidence on HSDD therapeutic approaches is largely reflective of short-term treatment effects in narrowly defined populations, essentially with limited insight into long-term efficacy and durability of response, or even broader patient applicability. Pharmacological treatment options for HSDD remain limited, with flibanserin and bremelanotide being the only agents to reach advanced clinical development. While both target central nervous system pathways, their efficacy remains modest and variable across trials. Current evidence highlights the need for novel therapeutic approaches and improved trial design to enhance clinical outcomes.

### Efficacy trends

Across trials, efficacy assessment centered on validated patient-reported outcome measures, particularly the FSFI desire domain and the FSDS (26, 27). The consistent use of these instruments, supports the growing consensus regarding the importance of measuring both sexual desire and associated distress (27). However, heterogeneity in endpoint designation and assessment timing, as well as reporting granularity, comes out as a major factor complicating cross-trial comparison and also serving to limit the ability to draw definitive conclusions regarding relative therapeutic benefit.

Registry-reported outcomes suggested modest and inconsistent efficacy across investigational agents, notably with some studies demonstrating improvements in sexual desire and reductions in distress relative to placebo while in others, findings reported more modest or inconsistent effects. Importantly, registry data often lacked detailed numerical results and this was a factor that made it difficult to evaluate effect sizes or clinical meaningfulness within the current study. It is also vital to point out here that this limitation reinforces the need for cautious interpretation of efficacy signals derived solely from registry entries. Importantly, this study does not permit conclusions regarding the comparative effectiveness of individual agents.

### Safety considerations

Safety and tolerability data were reported for most included trials; however, the depth and consistency of reporting varied substantially. Adverse events commonly reflected the centrally acting nature of the investigational agents and included neurological, gastrointestinal, and cardiovascular-related symptoms. On the overall, the reported adverse events were generally consistent with the known profiles of centrally acting agents. Serious adverse events were infrequently reported, and discontinuation rates due to adverse events were generally low. Long-term safety information was primarily derived from an open-label extension study, highlighting the scarcity of prolonged safety evaluations in HSDD pharmacotherapy (3). Given that sexual desire disorders often require chronic management (28), the limited availability of long-term safety and tolerability data as discovered in the current study does represent a notable gap in the current evidence base.

### Methodological and reporting limitations

Several methodological and reporting limitations identified in this analysis may affect the interpretation and generalizability of findings. Upfront, the relatively small number of included trials (*n* = 9) reflects the application of strict inclusion criteria and the limited number of completed interventional studies evaluating pharmacological therapies for HSDD within the registry during the study period. This reflects the strict eligibility criteria applied (completed, interventional, pharmacological trials in adult women with HSDD), as well as the limited number of advanced-stage pharmacological studies in this field. While there was a substantial number of trials showing up with some relevant content from the preliminary search, upon further review many of these were unsuitable for inclusion in this review. Notably, this aspect, then being a weakness in the current study, is consistent with the early-stage and narrowly focused nature of pharmacological development in HSDD, where only a limited number of agents have progressed to late-phase clinical evaluation. The diagnostic criteria and population definitions do vary remarkably across trials with inconsistent reporting of menopausal status, psychosocial factors, and comorbid conditions. Notably, these inconsistencies have the potential to contribute to variability in treatment response and limit generalizability. Incomplete result reporting within ClinicalTrials.gov emerged as another limitation in the current study, a challenge that constrained comprehensive evaluation of trial outcomes. Although trial registration enhances transparency, the absence of posted results for some completed studies can substantially limit the utility of registry-based analyses and raises concerns regarding selective dissemination of findings. This limitation is inherent to registry-based research and may introduce reporting bias, particularly when results are selectively posted. Accordingly, improved compliance with result reporting requirements is essential for the purpose of strengthening the evidence base and support informed clinical decision-making. Finally, the descriptive nature of this registry-based study precludes direct comparison of efficacy across agents or pooled quantitative analysis. While this approach is appropriate in consideration to the heterogeneity in study designs and outcome measures, it points to the need for more standardized trial methodologies in future research. In addition, and following these limitations, we recommend that while registry-based analyses as our study achieved provide valuable insights into research trends, there is a need to interpret them as complementary to, rather than a substitute for, detailed peer-reviewed evidence. Overall, this study highlights the need for greater standardization in trial design, endpoint selection, and reporting practices to improve the interpretability and clinical relevance of HSDD research.

### Clinical and research implications

From a clinical perspective, the findings illustrate both the promise and the limitations of currently investigated pharmacological treatments for HSDD. The emphasis on centrally acting agents reflects an improved understanding of the neurobiological underpinnings of sexual desire; however, modest efficacy signals and safety considerations highlight the importance of individualized treatment decisions and shared decision-making. For researchers and drug developers, the analysis identifies several priorities for future trials, including broader inclusion of postmenopausal women, longer follow-up periods, standardized outcome measures, and more comprehensive safety reporting. Incorporating patient-centered endpoints and real-world considerations may further enhance the relevance and impact of future studies. Flibanserin and bremelanotide represent the two most advanced pharmacological options for HSDD, but differ in their clinical use (29). Flibanserin is administered as a daily oral therapy and is more suitable for continuous treatment, whereas bremelanotide is used as an on-demand subcutaneous injection, offering flexibility for episodic use. Both agents demonstrate modest efficacy and have distinct safety profiles, which should be considered when selecting therapy.

### Future directions

In light of the above limitations and challenges, it is recommended that future clinical trials in HSDD should aim to expand beyond the narrowly defined populations and short-term efficacy assessments. More importantly, greater emphasis on long-term outcomes and comparative effectiveness, as well as real-world applicability is warranted. Additionally, it is the view of the authors here that exploring novel pharmacological targets and combination approaches harbors the potential to help address the unmet therapeutic needs in female sexual health. Previous systematic reviews have primarily focused on published clinical trials or individual pharmacological agents. In contrast, the present study provides a registry-based perspective, capturing both published and unpublished completed trials from ClinicalTrials.gov, thereby offering a broader view of the evidence base and highlighting gaps in reporting.

## Conclusion

In summary, the findings of this study strongly demonstrate that pharmacological research in HSDD is progressing, with a growing number of studies employing rigorous methodological designs. Investigational agents targeting diverse neurobiological pathways show variable but generally favorable efficacy and acceptable safety profiles. Nonetheless, incomplete reporting and heterogeneity in diagnostic criteria as well as variability in outcome measures, and limited long-term data, do remain significant challenges. As such, addressing these gaps through standardized reporting, longer follow-up and comparative studies will be essential to advance evidence-based management of HSDD and to optimize therapeutic outcomes for affected women.

## Data Availability

Publicly available datasets were analyzed in this study. This data can be found at: https://www.clinicaltrials.gov/.
